# Severe phenotypes of *B3GAT3-*related disorder caused by two heterozygous variants: a case report and literature review

**DOI:** 10.1186/s12920-022-01160-9

**Published:** 2022-02-12

**Authors:** Ying Li, Chuangwen Zhang, Hongyu Zhang, Weiqi Feng, Qiuji Wang, Ruixin Fan

**Affiliations:** 1 Department of Cardiac Surgery, Guangdong Cardiovascular Institute, Guangdong Provincial People’s Hospital, Guangdong Academy of Medical Sciences, Guangzhou, China; 2 Department of Cardiac Surgery, South China University of Technology, Guangzhou, China; 3 The Second School of Clinical Medicine, Southern Medical University, Guangzhou, China

**Keywords:** *B3GAT3*, Linkeropathy, Marfan syndrome, Cardiovascular defect, Aortic root dilation

## Abstract

**Background:**

Linkeropathies refers to a series of extremely rare hereditary connective tissue diseases affected by various glycosyltransferases in the biosynthesis of proteoglycans. We report for the first time two heterozygous variants of *B3GAT3* in a Chinese infant, in whom Marfan syndrome was suspected at birth.

**Case presentation:**

A 2-month-old boy from a non-consanguineous Chinese family without a family history presented severe phenotypes of joint dislocation, obvious flexion contractures of the elbow, arachnodactyly with slightly adducted thumbs, cranial dysplasia, foot abnormalities and aortic root dilation; Marfan syndrome was suspected at birth. Our patient was the youngest, at the age of 2 months, to experience aortic root dilation. Two *B3GAT3* variants, NM_012200.2, c.752T>C, p.V251A and c.47C>A, p.S16*, with heterozygosity were identified in the patient by whole-exome sequencing; the variants were inherited from his parents. During close follow-up, significant changes in the cranial profile and obvious external hydrocephalus were present at the age of 7 months, which differs from previously reported cases.

**Conclusion:**

We diagnosed a patient with congenital heart defects at an early age with a *B3GAT3*-related disorder instead of Marfan syndrome and expanded the spectrum of *B3GAT3*-related disorders. We also provide a literature review of reported *B3GAT3* cases; for at least one of the variants, this is the first report of genotype–phenotype correlations in individuals with cardiovascular defects being related to the acceptor substrate-binding subdomain of *B3GAT3.*

## Background

Linkeropathies, namely, a series of rare multisystem hereditary connective tissue diseases affected by abnormal biosynthesis of proteoglycan (PGs), feature a diversity of clinical manifestations, such as short stature, skeletal deformity, brachycephaly, joint contracture and/or dislocation, craniofacial abnormalities and cardiac defects. PGs in the extracellular matrix consist of one or more glycosaminoglycan (GAG) chains attached to core proteins, playing key roles in the growth and differentiation of cells as well as cell–cell and cell–matrix interactions [1]. Secreted PGs are responsible for the structure of cartilage and bone and are closely related to the development of linkeropathies [2]. The biosynthesis of the common tetrasaccharide linker region GlcUAβ1-3Galβ1-3Galβ1-4Xyl between the core protein and the hydrophilic glycosaminoglycan side chain of PGs is a complex multi-step process that involves five genes encoding various glycosyltransferases, including *XYLT1* (MIM: 608124), *XYLT2* (MIM: 608125), *B4GALT7* (MIM: 604327), *B3GALT6* (MIM: 615291), and *B3GAT3* (MIM: 606374). The beta1,3-glucuronyltransferase (GlcAT-I) encoded by *B3GAT3* completes the last step of transfer of a glucuronic acid (GlcA) from the donor substrate uridine diphosphate-glucuronic acid (UDP-GlcUA) to the linkage region Gal-β-(1-3)-Gal-β-(1-4)-Xyl [1, 3]. Variants in these five genes have been reported in patients with variable phenotypes of linkeropathies [4–6].

**Fig. 1:**
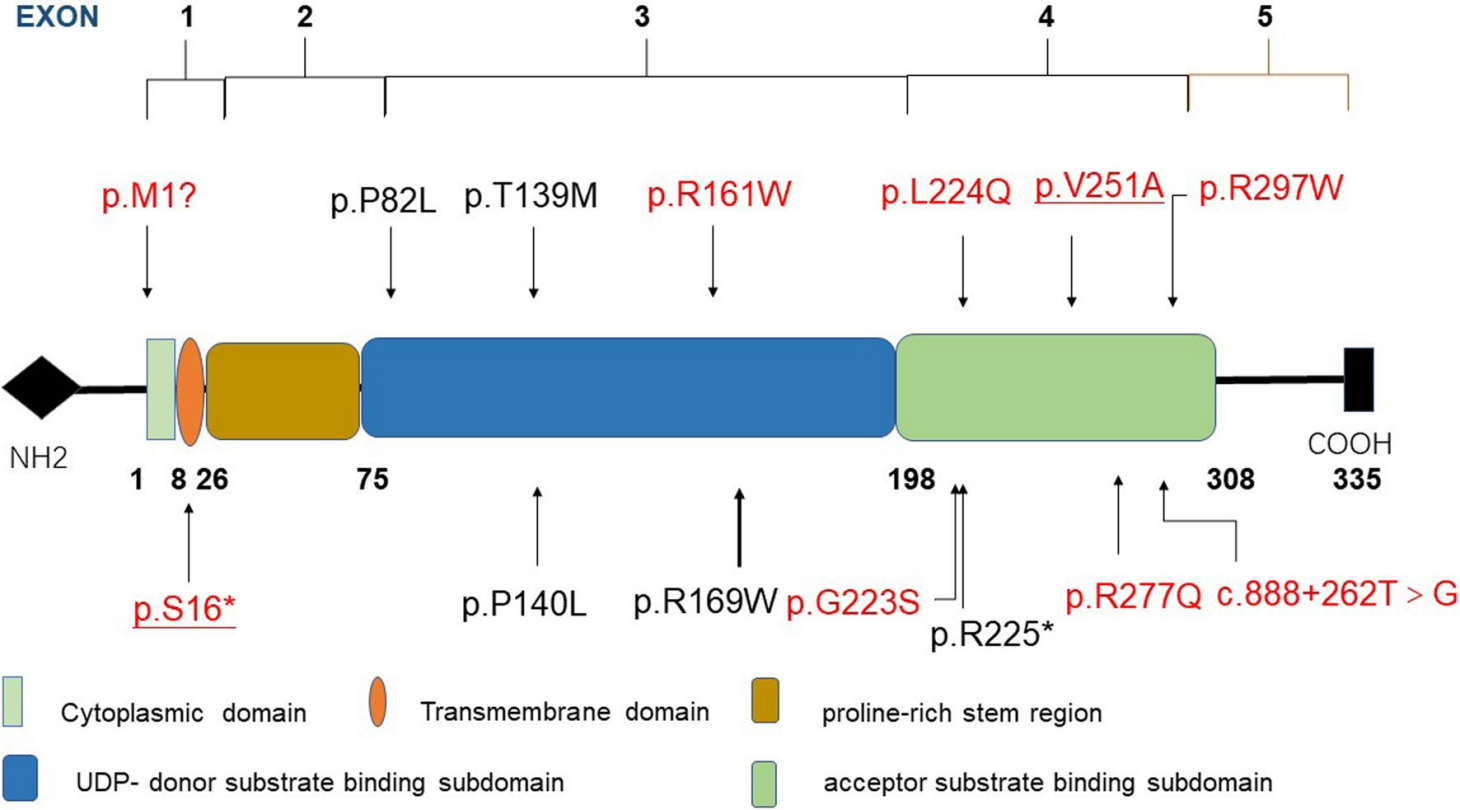
Structure of *B3GAT3* with all reported variants. Variants associated with a phenotype of cardiovascular abnormalities are marked in red; our patient is underlined

GlcAT-I contains 335 residues, including a transmembrane domain, cytoplasmic region, proline-rich stem region and catalytic region, with complicated components of donor and acceptor substrate binding subdomains at residues 75-197 and 198-308, respectively [7, 8]. *B3GAT3*-related disorders (*B3GAT3*-RD) are autosomal recessive diseases characterized by dislocations of the elbows, hips, and knees, foot deformities, short stature, and cardiovascular defects. It has been reported that pathological variants related to the phenotypes of *B3GAT3*-RD are mainly located in the catalytic region, with substrate acceptor-binding subdomain variants tending to be associated with more severe phenotypes; conversely, donor-binding subdomain variants lead to milder phenotypes [9]. To date, 12 variants in the *B3GAT3* gene have been described in 29 patients from 15 families with mild to severe phenotypes, but only half of them presented with heart defects (Fig. 1).

Here, we report a 7-month-old boy with two heterozygous variants located in the acceptor substrate-binding subdomain and transmembrane domain of GlcAT-I from a nonconsanguineous healthy family in China, in whom Marfan syndrome (MFS, MIM: 154700) was suspected at birth. We also provide a literature review of clinical and molecular data in reported *B3GAT3*-RD patients displaying cardiovascular defects, which expands the phenotypic spectrum of *B3GAT3-*related disorders with cardiovascular defects.

## Case presentation

**Fig. 2:**
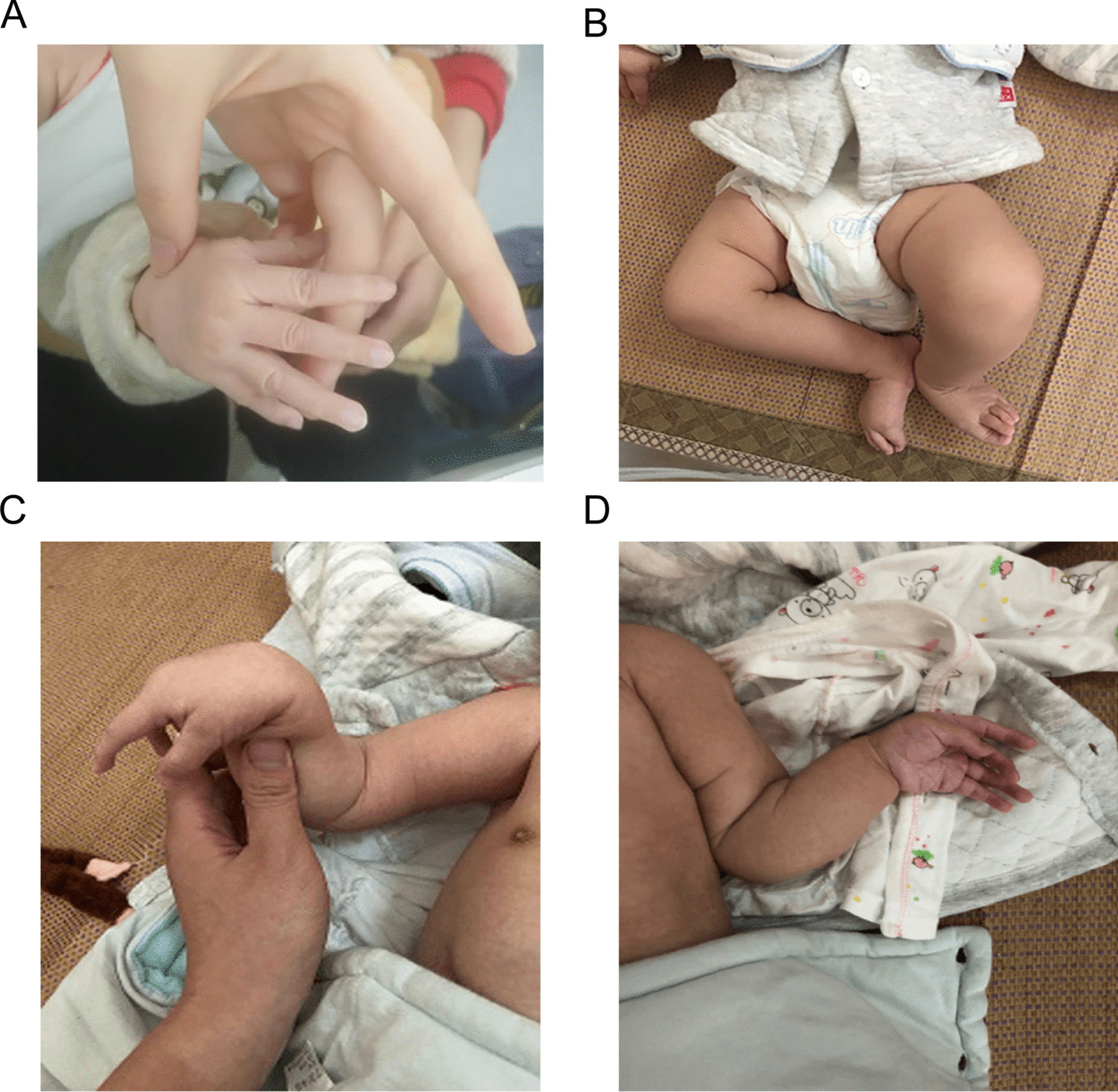
Clinical manifestation in this proband. **A** Long fingers at age of 2 months. Clinical features at the age of 5 months; **B** foot abnormality; **C** joint flexion contracture in the right elbow, long fingers with slightly adducted thumbs; **D** left cubitus valgus

The proband boy was born in a non-consanguineous Chinese family without a history of hereditary diseases. He was taken to the Guangdong Provincial People’s Hospital in Guangdong, China at 2 months of age for genetic counselling with a discharged diagnosis of suspected Marfan syndrome at birth due to arachnodactyly. He was the only baby of this Chinese family. Both parents were healthy, without a history of analogous occurrence. The pregnancy had gone well until the occurrence of gestational diabetes mellitus and decreased amniotic fluid in the last trimester. Although maternal blood glucose was well managed with diet, the amniotic fluid decreased over time. Figure 2 shows the clinical characteristics of this proband. Clinical follow-up occurred at the ages of 5 and 7 months separately. Radiological examination was also performed. We obtained permission to publish the pictures and clinical data.

As mentioned above, the pregnant mother developed gestational diabetes mellitus in the third trimester that was well controlled by diet; a decrease in amniotic fluid was noticed at 30 plus weeks. The amniotic fluid index (AFI) was 7.5 cm at 38 weeks of gestation, whereas the AFI was reduced to 7.1 cm after 7 days. Consequently, caesarean section was induced at 39+ weeks of gestation due to oligohydramnios. The newborn baby survived and was in good condition, with a weight of 3000 g; the Apgar score was 10 points both at 1 min and 10 min. However, the child presented with long fingers and toes that were highly suspected to be caused by MFS. At the age of 2 months, he had a widened forehead, depressed nasal bridge, blue sclera, short neck, arachnodactyly and long toes. Echocardiography showed an increase in the diameter of the aortic sinus of 12.6 mm, but the arch of the aorta and the descending aorta were normal. When assessing development of the limbs at the age of 5 months, we discovered obvious flexion contractures of the right elbow, scoliosis, long fingers with slightly adducted thumbs, foot abnormality, and body length that was 66 cm, longer by two centimetres than the previous month, and a condition of bent limbs. The occipitofrontal circumference was 45 cm at the age of five and a half months. His cutaneous features showed reticular marble skin, especially over the thoracoabdominal skin and dorsum of hands, without any cutis laxa-like appearance, as occurs in others [9, 10].

**Fig. 3:**
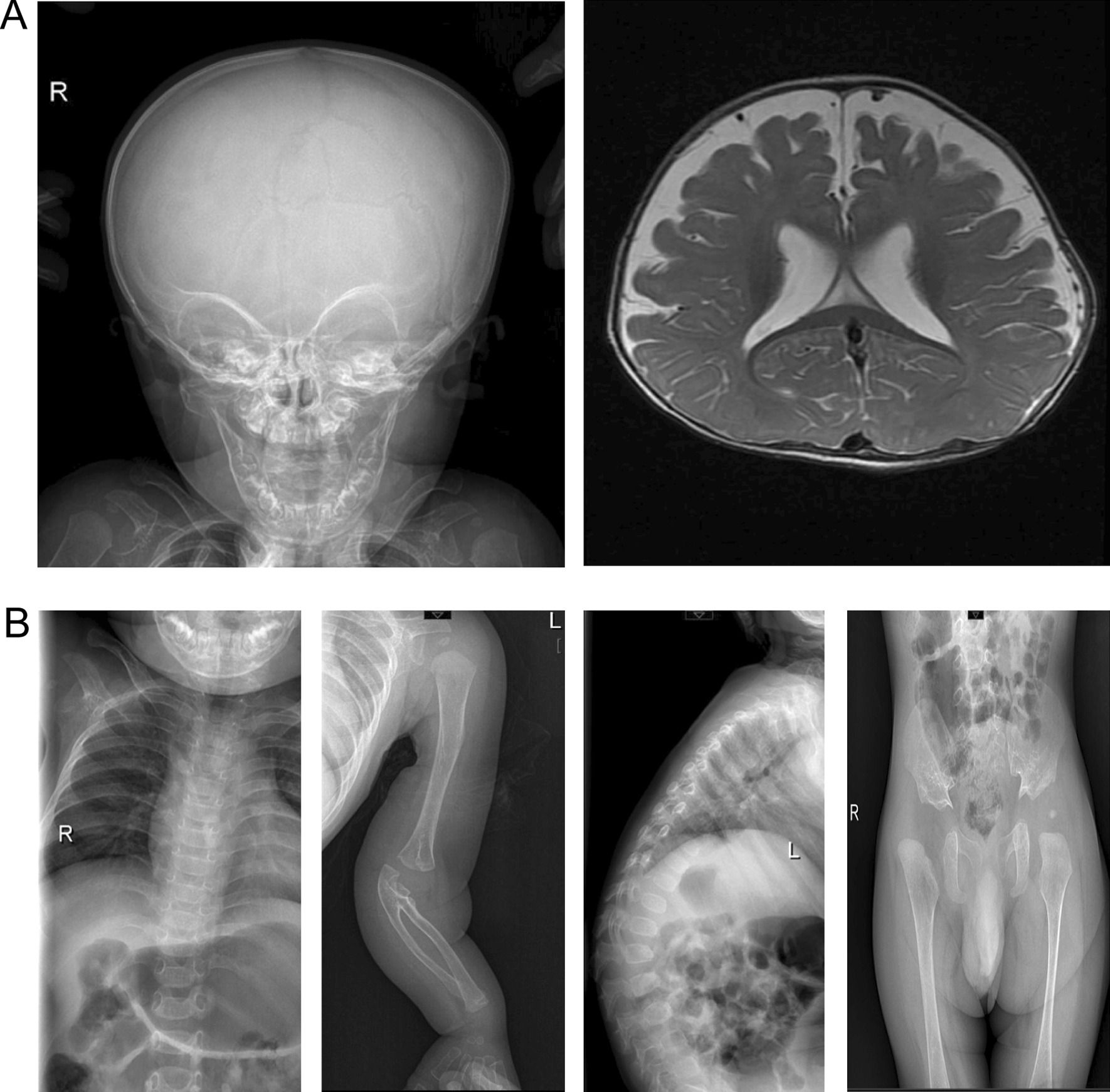
Clinical and radiological features in the present patient at the age of 7 months. Radiological features: **A** enlarged head, external hydrocephalus on MRI, **B** Scoliosis, left elbow dislocation, radioulnar synostosis, vertebral instability on T11, and T12, and bilateral dislocation of the hip

**Fig. 4:**
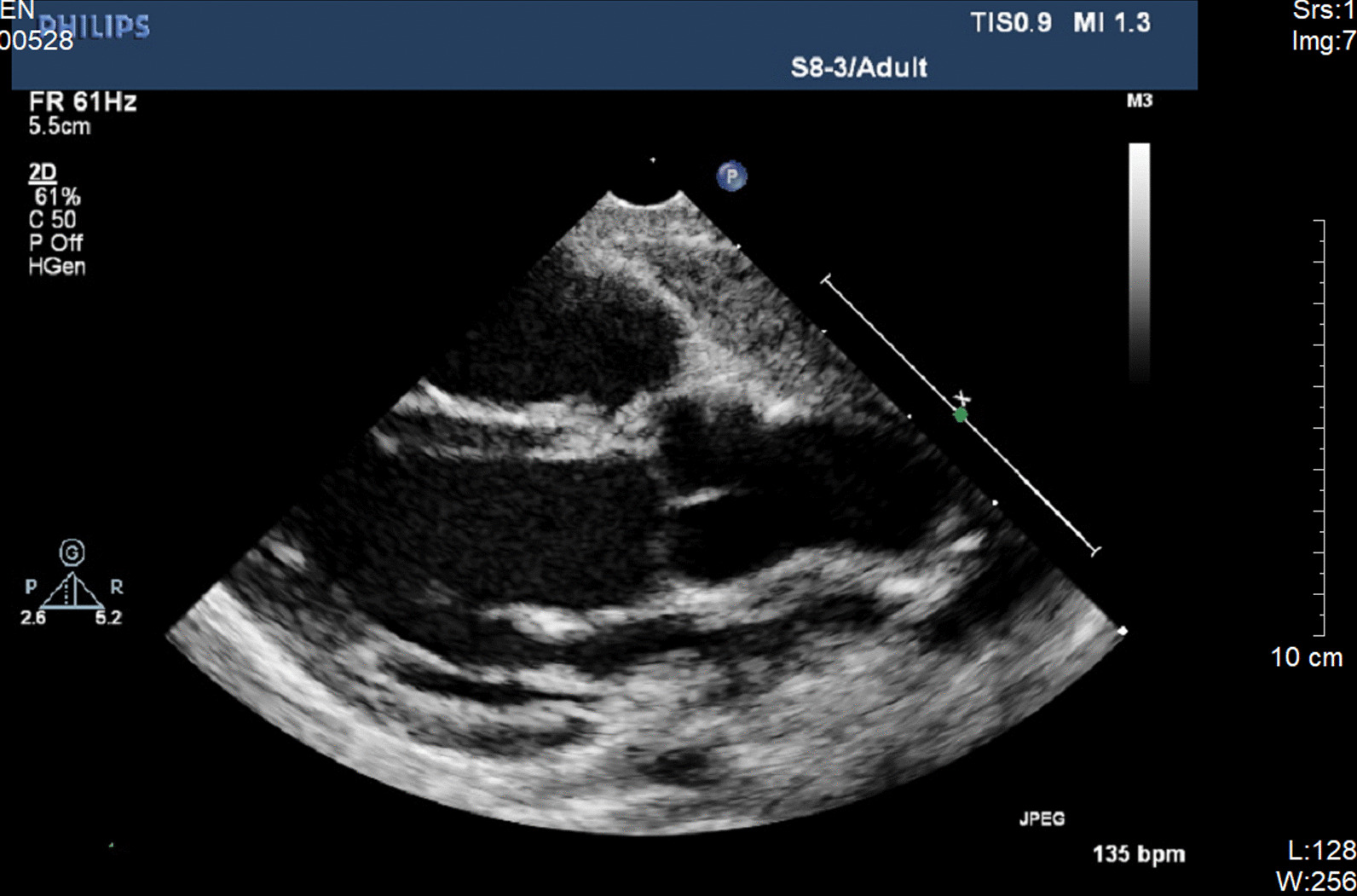
Echocardiography showing a broadened sinus of aorta

MRI was performed at the age of 7 months due to macrocephalus and a large anterior fontanel, indicating enlarged cisterns and frontotemporal sulcus. He was diagnosed by a neurosurgeon with external hydrocephalus, which could seriously affect the intellectual development and lack of available therapeutic measures. Dislocated joints of the left elbow, bilateral hips and vertebral instability of T11 and T12 were also noted by radiological examination. Symptomatic treatment and follow-up were suggested by clinicians. The clinical features and examination of this patient at the age of 7 months are shown in Fig. 3. The diameter of the aortic sinus was 16 mm, with a Z-score of 2.55 (Fig. 4).

**Fig. 5:**
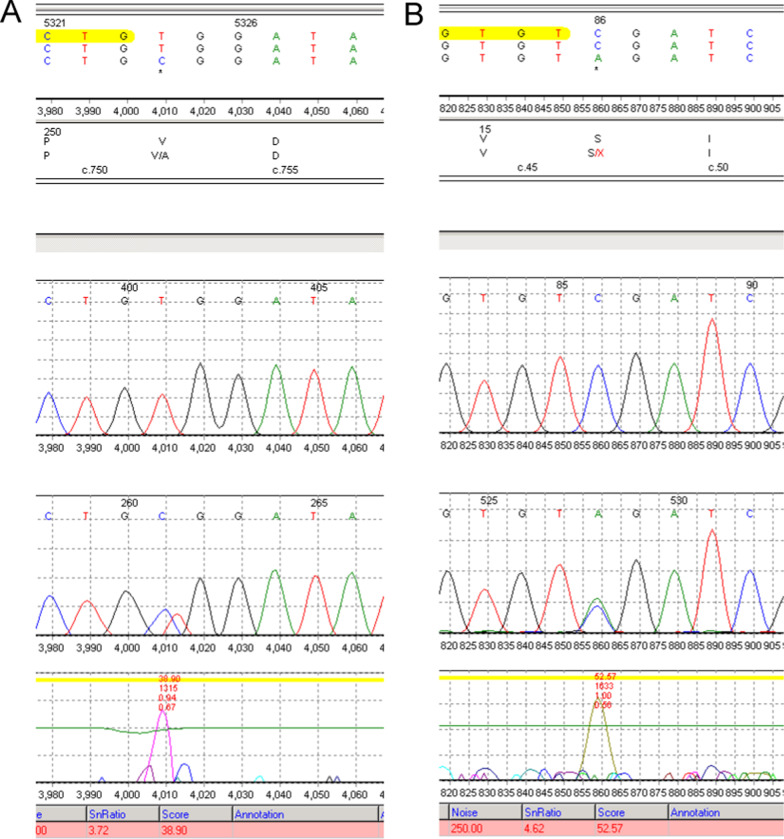
Sanger sequencing revealing c.752T>C (p.V251A) (**A**) and c.47C>A (p.S16*) (**B**)

Informed consent for whole-exome sequencing using peripheral blood from the proband and his parents was obtained. Whole-exome sequencing analysis identified two heterozygous variants in the *B3GAT3* gene, c.752T>C (p.V251A) and c.47C>A (p.S16*), which was confirmed by Sanger sequencing. (Table 1, Fig. 5) The paternally inherited p.V251A variant, classified as VUS (variant of uncertain significance) according to the American College of Medical Genetics guidelines [11], has low frequencies (< 0.001) in population genomic databases and is predicted to be a “damaging” variant by SIFT (0.003) and Polyphen2-HDIV (0.725). The maternally inherited S16* variant is a likely pathogenic variant. No variants in the MFS causative genes of *FBN1, ACTA2, MYLK,* or *MYH11* were identified, indicating that Marfan syndrome was determined to be less likely. Thus, the patient was diagnosed with *B3GAT3*-related disorder.

**Table 1:** Compound heterozygous variant of *B3GAT3* in our patient

Location of HG19	Variant	Frequencies	Classification of ACMG	Origin
Chr11:62384135	c.752T>C, p.V251A	< 0.001	VUS	Paternal (heterozygote)
Chr11:62389373	c.47C>A, p.S16*	–	LP	Maternal (heterozygote)

*VUS* variant of uncertain significance, *LP* likely pathogenic

## Discussion and conclusions

Herein, we identify two *B3GAT3* variants with heterozygosity in a Chinese family; this is the fifth patients being compound heterozygous reported in the literature thus far (shown in Table 3), differing from others with homozygous variant.

*B3GAT3*-related disorder is an extremely rare disease characterized by various manifestations varying from mild to severe. To date, only 30 patients with 14 variants in *B3GAT3* have been reported in 16 families from different countries [9, 10, 12–20], including our patient; among cases, skeletal dysplasia and facial deformity are common, whereas cardiovascular defects only affected 14 individuals (Table 2). Among them, the most common cardiovascular abnormalities are aortic root dilation and ventricular septal defect (in 6 patients), bicuspid aortic valve (BAV; in 4), valve insufficiency, including the—mitral, tricuspid and aortic valves (—in 5), patent foramen ovale (PFO; in 5), atrial septal defect (ASD; in 3), ascending aorta dilation, patent ductus arteriosus (PDA) and pulmonary stenosis (in 1). Our patient was the youngest to display aortic-related events, namely, at the age of 2 months.

**Table 2:** Clinical characteristics of all reported individuals with *B3GAT3* variants

References	[12]	[13]	[14]	[19]	[10]	[15]	[16]	[17]	[18]	[9]	[20]	Our patient	Total
Year	2011	2014	2015	2015	2016	2016	2017	2018	2019	2019	2020		
Variant	c.830G>A p.R277Q	c.830G>A p.R277Q	c.419C>T p.P140L	c.667G>A p.G223S	c.245C>T p.P82L	c.1A>G, p.M1? c.671T>A, p.L224Q	c.888+262 T>G	c.667G>A p.G223S	c.481C>T, p.R161W c.889C>T p.R297W	c.667G>A, p.G223S c.416C>T, p.T139M	c.505C>T p.R169W c.673C>T, p.R225*	c.752T>C, p.V251A c.47C>A, p.S16*	14
Type	Homo	Homo	Homo	Homo	Homo	Compound hetero	Hetero	Homo	Compound hetero	Homo	Compound hetero	Compound hetero	–
Number	5	1	8	1	1	1	1	6	1	2	2	1	30
Nationality/ethnicity	Arab	Arab	Indonesian	Mexican	NR	Caucasian	Caucasian	Moroccan	Italian	Indian; Turkish	Australian	Chinese	/
Consanguineous family	+	+	+	+	NR	+	−	+	−	+	−	−	24
*Cardiovascular defects*	+	+	−	+	NR	+	+	3/6	+	0/1	−	+	14
ArD	3/5	+ (5y)	−	0/1	NR	+ (6y)	−	−	−	−	−	+ (2 m)	6
AoD	−	−	−	−	NR	+ (6y)	−	−	−	−	−	−	1
ASD	1/5	−	−	+	NR	−	−	−	+ (2y)	−	−	−	3
VSD	2/5	−	−	+	NR	−	+	2/6	−	−	−	−	6
PDA	−	−	−	+	NR	−	−	−	−	−	−	−	1
BAV	3/5	−	−	−	NR	+ (2w)	−	−	−	−	−	−	4
Valve insufficiency	4/5	−	−	−	NR	−	−	−	+	−	−	−	5
PFO	3/5	−	−	−	NR	+ (2w)	−	−	−	−	−	+	5
PS	−	−	−	−	NR	−	+	−	−	−	−	1	1
Joint contracture	+	+	4/8	+	NR	NR	NR	+	−	1/2	1/1	+	20
Restricted elbow movement	NR	+	NR	+	NR	NR	NR	NR	−	−	NR	+	3
Joint dislocation	+	+	+	−	NR	+	−	3/6	+	1/2	+	+	23
Radioulnar synostosis	NR	+	2/2	+	NR	−	NR	+	+	NR	NR	+	12
Hypotonia	NR	−	NR	+	NR	+	NR	−	+	NR	NR	+	4
Kyphosis and /or scoliosis	0/3	−	4/8	−	+	+	NR	1/6	+	1/2	1/1	+	11
Long fingers and /or toes	NR	−	−	+	NR	+(6y)	NR	4/6	+	+	NR	+	10
Foot abnormalities	+	+	6/8	+	NR	−	NR	+	+	+	+	+	25
Cephalus quadratus	NR	NR	NR	NR	NR	NR	NR	NR	NR	NR	NR	+	1
External hydrocephalus	NR	NR	NR	NR	NR	NR	NR	NR	NR	NR	NR	+	1
Prominent /Wide forehead	NR	+	NR	+	NR	NR	+	NR	+	−	0/1	+	5
Blue sclerae	NR	−	NR	+	NR	+	NR	NR	+	1/2	1/1	+	6
Depressed nasal bridge	4/5	+	4/8	+	NR	−	NR	2/6	−	1/2	1/1	+	15
Short/webbed neck	+	+	2/8	+	NR	+	NR	NR	+	+	1/1	+	15
Cutis laxa	NR	−	NR	−	+	NR	NR	NR	−	1/2	NR	−	2
Hyperextensible skin	NR	−	−	NR	NR	+	NR	NR	+	−	NR	+	3
Motor developmental delay	−	+	NR	+	NR	+	NR	NR	+	1/1	NR	+	6
Intelligence disability	−	+	−	+	NR	+	NR	NR	−	0/1	/	+	4
Fractures	NR	NR	NR	+	+	+	−	4/6	−	1/2	−	−	8

*Homo* homozygous, *hetero* heterozygous, *compound hetero* compound heterozygosity, *ArD* aortic root dilation, *AoD* ascending aorta dilation, *ASD* atrial septal defect, *VSD* ventricular septal defect, *PDA* patent ductus arteriosus, *BAV* bicuspid aortic valve, *PFO* patent foramen ovale, *PS* pulmonary stenosis, *NR* not reported, *Na* not available

Baasanjav et al. for the first time reported Larsen-like syndrome with variable malformations of the heart with *B3GAT3* variants, including BAV, aortic root dilation, mitral valve prolapse, ASD and PFO, and demonstrated expression of *B3GAT3* RNA in the heart, aorta, bone and osteoblasts of mouse tissues and the presence of GlcAT-I protein in the human aorta [12]. Correspondingly, almost half of individuals with *B3GAT3* variants have been reported to present heart defects, half of whom developed aortic diseases such as aortic root dilation and ascending aorta dilation (shown in Table 2). Very recently, Colman et al. [9] concluded that the two subdomains of the catalytic region of GlcAT-I are related to the phenotypes, despite only limited reports. Moreover, the more severe phenotypes tended to be associated with variants in the substrate acceptor—binding subdomain, whereas donor-binding subdomain variant manifest as milder phenotype. To date, five patients being compound heterozygous have been reported, among whom at least one variant is located in the substrate acceptor—binding subdomain. (Table 3, Fig. 1) [15, 18, 20, 21]. Among individuals with compound heterozygous variants in *B3GAT3*, our patient was the youngest to exhibit defined heart defects, and an enlarged sinus of the aorta is severely associated with an increased risk of death and cardiovascular complications, especially in neonates.

**Table 3:** Summary of five patients with compound heterozygosity in *B3GAT3*

Nationality/race/ethnicity	Consanguineous family	Age	Sex	Variant	Classification	Exon	Domain
Caucasian	Yes	4y	M	c.1A>G, p.M1?	LP	1	Cytoplasmic domain
				c.671T>A, p.L224Q	VUS	4	Acceptor substrate binding subdomain
Italian	No	13y	F	c.481C>T, p.R161W	LP	3	Donor substrate binding subdomain
				c.889C>T, p.R297W	LP	4	Acceptor substrate binding subdomain
Australian	No	Stillborn at 16 w	M	c.505C>T, p.R169W	D*	3	Donor substrate binding subdomain
				c.673C>T, p.R225*	LP	4	Acceptor substrate binding subdomain
		Deceased at 9m	F	c.505C>T, p.R169W	D*	3	Donor substrate binding subdomain
				c.673C>T, p.R225*	LP	4	Acceptor substrate binding subdomain
Chinese	No	2m	M	c.752T>C, p.V251A	VUS	4	Acceptor substrate binding subdomain
				c.47C>A, p.S16*	LP	1	Transmembrane domain

*y* year, *m* month, *w* week, *Male* M, *Female* F, *LP* likely pathogenic, *VUS* variant of uncertain significance, *D* predicted to be deleterious by multiple lines of computational evidences

*Not an ACMG classification

Based on Pedersen’s research about the structure of *B3GAT3* [8], we identified that among patients with cardiovascular abnormalities, at least one variant in the acceptor substrate-binding subdomain of *B3GAT3* was detected, which seems to be related to a severe phenotype (shown in Fig. 5). Variant of p.G223S occurred in six families, but only half of them presented with cardiovascular defects due to phenotypic disparity. Two patients with compound heterozygous variants, including p.R225*, exhibited more severe manifestations and lethality, without any heart defects [20]. Further insight into the relationship between altered GlcAT-I and heart defects is needed due to the limited data.

It is interesting to note that patients harboring the same variant display variable phenotypes. p.R277Q was detected in 6 patients of 2 unrelated consanguineous Arab families [12, 13]. In contrast to the five siblings in the first large family, a 5-year-old boy developed distinct manifestations of dental abnormalities, refractive error, skin wrinkling, developmental delay, and bilateral inguinal hernia. Similarly, the same p.G223S variant with extremely severe phenotypes occurred in 8 subjects of 6 families from different countries, most of whom died before the age of 1 year [9, 17, 19]. The clinical manifestations are not exactly the same, except for common symptoms of radioulnar synostosis, joint contractures, and foot deformity. More data are needed to determine whether there is a “hotspot” variant in *B3GAT3*.

Overlapping and diverse features of various syndromes make diagnosis difficult. Six patients suspected of having Antley–Bixler syndrome with prenatal craniosynostosis were eventually diagnosed with *B3GAT3*-RD by genetic testing. In the present study, based on arachnodactyly and skeletal dysplasia at birth and an enlarged aortic sinus at the age of 2, the infant was suspected of having neonatal Marfan syndrome (nMFS) [22–25], which is the most severe form of MFS. MFS is a group of rare hereditary connective tissue diseases caused by the fibrillin-1 gene (*FBN1* [MIM: 134797]), featuring skeletal deformity, pectus excavatum, arachnodactyly, long, narrow face, malar hypoplasia, micrognathia, retrognathia, ocular disorder and cardiovascular abnormalities including aortic root dilation, and mitral or tricuspid insufficiency [26],—whereas nMFS mainly occurs in infants and young children. The prognosis of nMFS is so poor that it has extremely high mortality before the age of 2 years [27] and the most patients die from rapid progression of dilation of the aorta. Exons 24–32 are known as the neonatal region of *FBN1*, leading to a relative genotype–phenotype correlation in nMFS [28, 29] For our case, after performing whole-exome sequencing, we think it less likely to be diagnosed as MFS due to the lack of *FBN1* variant. In addition, Loeys–Dietz syndrome (*LDS4* [MIM: 614816], *LDS5* [MIM: 615582]), Ehlers–Danlos syndrome (*EDS* [MIM: 130000]), and Beals syndrome (MIM: 121050) may share some specific phenotypic features of skeletal deformity and cardiovascular abnormalities [30, 31]. Therefore, identifying the cause of hereditary diseases as early as possible and making correct differential diagnoses by gene sequencing are conducive to clinical care and treatment.

The birth prevalence of congenital anomalies is estimated to be 20.8 per 1000 registered births [32];—congenital heart diseases (CHDs) are the most common, with figures varying from 2.5 to 17 per 1000 live births [33], and closely related to perinatal and infant mortality. Complex conditions comprise only ~ 15% of congenital heart diseases with a clearly known cause, while syndromic CHD with single gene variants accounts for 3–5% of congenital heart defects, such as Holt-Oram syndrome(MIM: 142900) and Noonan syndrome (NS4 [MIM: 610733]) [34]. In addition, Larsen-like syndrome caused by *B3GAT3* variant involves congenital heart defects as well as skeletal dysplasia, increasing the risk of death. Maternal diabetes mellitus is one of the main causes of CHD, in which the accumulation of metabolic fuels affects the most fundamental process of cardiac development through altered gene expression [35]. In fact, we found that two pregnant mothers (including our presentation) developed gestational diabetes mellitus that was well controlled by diet during pregnancy; both sons presented variable cardiovascular defects with compound heterozygosity in *B3GAT3* [15].

In conclusion, we report a 7-month-old boy with cardiovascular defects at an early age who carried two heterozygous variants in *B3GAT3* and in whom MFS was suspected. The findings contribute to the spectrum of congenital cardiovascular abnormalities in *B3GAT3*-RD, although heart defects are not present in every case.

## Data Availability

The raw sequence data reported in this paper have been deposited in the Genome Sequence Archive (Genomics, Proteomics & Bioinformatics 2021) in National Genomics Data Center (Nucleic Acids Res 2021), China National Center for Bioinformation / Beijing Institute of Genomics, Chinese Academy of Sciences, under accession number HRA001274 that are publicly accessible at https://ngdc.cncb.ac.cn/gsa-human.

## Abbreviations

PG: Proteoglycan
GAG: Glycosaminoglycan
B3GAT3: Beta-1,3-glucuronyltransferase
MFS: Marfan syndrome
AFI: Amniotic fluid index
nMFS: Neonatal Marfan syndrome
LDS: Loeys–Dietz syndrome
EDS: Ehlers–Danlos syndrome
CHD: Congenital heart diseases
NS: Noonan syndrome
BAV: Bicuspid aortic valve
PFO: Patent foramen ovale
ASD: Atrial septal defect
PDA: Patent ductus arteriosus
